# In Silico Approach for Immunohistochemical Evaluation of a Cytoplasmic Marker in Breast Cancer

**DOI:** 10.3390/cancers10120517

**Published:** 2018-12-15

**Authors:** Claudia Mazo, Estibaliz Orue-Etxebarria, Ignacio Zabalza, Maria d. M. Vivanco, Robert M. Kypta, Andoni Beristain

**Affiliations:** 1 Vicomtech, eHealth and Biomedical Applications, 20009 San Sebastian-Donostia, Spain; aberistain@vicomtech.org; 2 School of Computer Science, University College Dublin, D14 YH57 Dublin, Ireland; 3 CeADAR: Centre for Applied Data Analytics Research, D04 V1 W8 Dublin, Ireland; 4 CIC bioGUNE, Center for Cooperative Research in Biosciences, 48160 Bilbao, Spain; eorue@cicbiogune.es (E.O.-E.); mdmvivanco@cicbiogune.es (M.d.M.V.); rkypta@cicbiogune.es (R.M.K.); 5 Department of Pathology, Galdakao-Usansolo Hospital, 48960 Galdakao, Spain; ignacio.zabalzaestevez@osakidetza.eus; 6 Imperial College London, Department of Surgery and Cancer, London SW7 2AZ, UK

**Keywords:** breast cancer, *Wnt-1*, immunohistochemistry (IHC), automatic segmentation, automatic quantification

## Abstract

Breast cancer is the most frequently diagnosed cancer in women and the second most common cancer overall, with nearly 1.7 million new cases worldwide every year. Breast cancer patients need accurate tools for early diagnosis and to improve treatment. Biomarkers are increasingly used to describe and evaluate tumours for prognosis, to facilitate and predict response to therapy and to evaluate residual tumor, post-treatment. Here, we evaluate different methods to separate Diaminobenzidine (DAB) from Hematoxylin and Eosin (H&E) staining for *Wnt-1*, a potential cytoplasmic breast cancer biomarker. A method comprising clustering and Color deconvolution allowed us to recognize and quantify *Wnt-1* levels accurately at pixel levels. Experimental validation was conducted using a set of 12,288 blocks of m×n pixels without overlap, extracted from a Tissue Microarray (TMA) composed of 192 tissue cores. Intraclass Correlations (ICC) among evaluators of the data of 0.634, 0.791, 0.551 and 0.63 for each Allred class and an average ICC of 0.752 among evaluators and automatic classification were obtained. Furthermore, this method received an average rating of 4.26 out of 5 in the *Wnt-1* segmentation process from the evaluators.

## 1. Introduction

Breast cancer is one of the most common types of cancer in most European and American countries and the second leading cause of cancer death [1,2,3]. About 266,120 new cases of invasive breast cancer are diagnosed in women in Europe every year. Breast cancer represents almost a sixth (16%) of all cases of cancer in males and females combined. However, mortality rates have decreased as a result of earlier diagnosis and improved therapies, according to the American Cancer Society and International Agency of Research Cancer [4]. The *WNT* genes encode a family of 19 secreted short-range signalling proteins involved in the regulation of cell fate, proliferation, migration, polarity and death, processes that play important roles in cancer initiation and/or progression [5]. Increased expression of the Cancer Stem Cell (CSC) marker *SOX2* in human breast cancer activates *Wnt-1* signaling to promote resistance to tamoxifen, the most common therapy for Estrogen Receptor-positive (ER+) breast cancer [6]. There are nineteen *Wnt-1* family members, several of which may play a role in breast cancer. Among them is *Wnt-1*, which was originally named int-1 because integration of the Mouse Mammary Tumor Virus (MMTV) into its gene locus induces mammary tumors in mice [7]. Consistent with this, *Wnt-1* increases the CSC population [8] and shRNA-mediated *Wnt-1*1 silencing in a metastatic mouse breast cancer cell line reduces expression of CSC markers and tumor formation [9]. There are fewer studies of *Wnt-1* in human breast cancer. However, *Wnt-1* protein has been detected in tumors of Korean patients with invasive ductal carcinoma [10] and has been reported to be upregulated in human breast tumors, compared with adjacent normal tissue [11]. Together, these data suggest that *Wnt-1* could be a useful biomarker and therapeutic target in breast cancer. In addition, *Wnt-1* expression may be an indicator of poor prognosis in cutaneous squamous cell carcinoma [12] and in non-small cell lung cancer [13], and a potential biomarker of recurrence in Hepatitis C-related hepatocellular carcinoma [14]. Although *Wnt-1* is a secreted protein, *Wnt-1* in the extracellular space is undetectable by immunohistochemistry, as is the case for most secreted proteins, which are more readily detected in the cytoplasm, being enriched in organelles of the secretory pathway. In this study, we therefore used *Wnt-1* as an example of a cytoplasmic marker to test our in silico method.

The classification and quantification of biomarker expression levels is carried out by pathologists, biologists and related professionals on a limited region of a tumor. Manual scoring is normally carried out by at least two scientists with experience in scoring the antigen, and at least including one pathologist. In this study, immunoreactivity for *Wnt-1* was scored using a semi-quantitative evaluation based on the Allred system [15]. An in silico approach may help to improve the reproducibility of scoring among pathologists, which is common according to the subjectivity of pathological diagnosis. It may also increase the number of cases that an expert can analyze. Thus, automated scoring will improve patient quality of life and reduce associated health-care costs. Additionally, this type of approach may be being helpful for the purpose of medical education [16]. Furthermore, an in silico approach could provide support to methods for discovering new biomarkers and mitigating issues related to inter-observer variability (e.g., bias, time, difficulty, costs, and impracticality) [17].

Some in silico approaches can recognize biomarkers—e.g., Estrogen Receptor (ER), Progesterone Receptor (PR), Phosphatase and Tensin Homolog (PTEN), Human Epidermal Growth Factor Receptor 2 (HER2), and *Ki-67*—in histopathology images [18,19,20,21,22]. A free ImageJ plugin and Internet-based web application, called ImmunoRatio, for automatic segmentation and quantification of ER, PR, and Ki-67 immunohistochemistry (IHC) in breast cancer tissue sections is presented in [18]. Color deconvolution, adaptive thresholding and median filter are used in ImmunoRatio. This approach achieved a correlation coefficient of 0.98. An important aspect to take into account to generate in silico approaches is the difference between visual assessment and automated methods. In [20], a comparison between visual assessment and Automated Digital Analysis (DIA) of Ki-67 is presented. That method demonstrates agreement between manual expert assessment—by eye—and DIA of Ki67 in breast cancer. However, the datasets for these studies are not publicly available, limiting their reproduction and comparison with similar approaches. A method to segment and characterize cells in carcinoma images stained with Ki-67 using K-means clustering and the watershed algorithm is proposed in [19]. The method calculates 75.1±6.7% positive nuclei compared with ImmunoRatio, 77.9±7.1%, and manual, 71.4±5.5%. However, this approach has not been tested on breast cancer images and the dataset is not publicly available. Furthermore, the aforementioned studies focus on Ki-67, which is a nuclear biomarker, rather than a cytoplasmic biomarker. In [21], an Automated Quantitative Analysis (AQUA) of biomarker expression detection with cytoplasmic or nuclear staining is described. This approach has been validated in tissue microarrays, as well as in whole tissue sections using images from ovarian and breast cancer [23]. However, AQUA employs multiplexed fluorescent stains to compartmentalize and measure expression of specific biomarkers and the image data are not publicly available. Bankhead et al. [22] describe an open-source solution for digital pathology and whole slide image analysis and employed annotation, segmentation, detection and classification using machine learning techniques.

Advances have been made by our group in the classification of healthy tissues and organs using histological images, for example using Masson’s trichrome and H&E [24]. Image processing techniques, tissue and organ morphological information, image features—color and texture—clustering, supervised learning and deep learning to identify fundamental tissues [25,26,27,28] and organs [29,30,31] have been used in our approach.

In this paper, we present an in silico approach to classify and quantify IHC staining of the predominantly cytoplasmic marker, *Wnt-1*, in breast cancer. We used color deconvolution, K-means algorithm and Allred score [32] to recognize and quantify *Wnt-1* levels. We propose that in silico methods such as this have a unique advantage of being able to reduce subjectivity and optimize visual scoring in greater detail.

This paper is structured as follows: the problem statement is presented in Section 4.1. The proposed approach to automatic classification and quantification of *Wnt-1* expression is explained in detail in Section 4. The dataset, the experiments and the results are described in Section 2. In Section 3, the study is discussed. Finally, the main conclusions of this work are drawn in Section 5.

## 2. Experiments and Results

**Figure 1:**
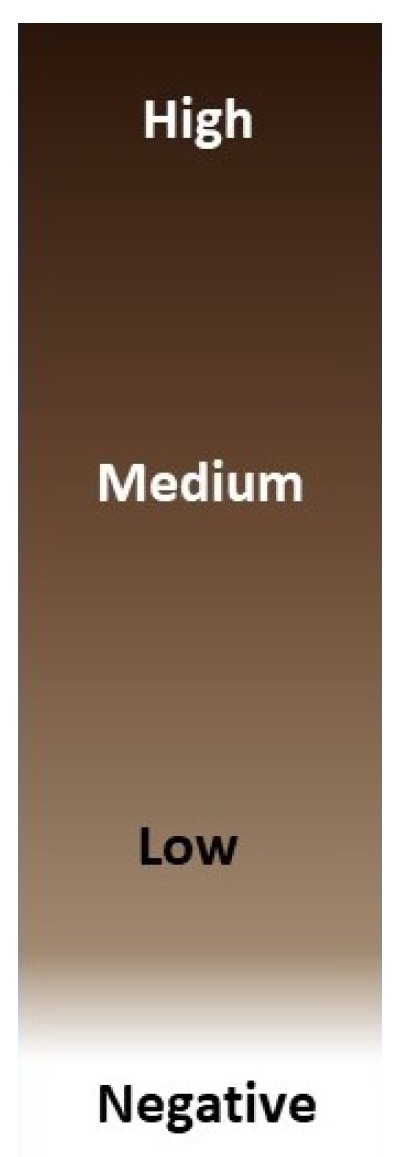
Labels and corresponding description for each one of the classes considered according to *Wnt-1* intensity at pixel levels, four quarterlies are identified.

In this section, we present the complete process to evaluate the proposed approach. We show the results obtained in three subsections: (i) block-based *Wnt-1* segmentation; (ii) block-based *Wnt-1* classification; and (iii) *Wnt-1* classification in a TMA tissue core image. Some measurements were calculated through subjective evaluation of a group by two experts of the research laboratory (R.K. and E.O.). The human is the best judge to evaluate the output of the segmentation algorithm, owing to the difficulty of obtaining ground truth for real images [33]. Many segmentation methods are assessed according to expert criteria [34]. Appealing to human intuition was convenient in our case, since our goal was to create a large dataset that can later be used to train our automated system. Taking each TMA tissue core image into account and the criteria defined using the Allred score, *Wnt-1* expression was classified as follows: (i) proportion of positive cells based on five levels—1=(0%,1%], 2=(1%,10%], 3=(10%,33%], 4=(33%,66%], and 5=(66%,100%], where the parentheses indicate an open interval that does not include its endpoints, and the square brackets indicate a closed interval that includes its endpoints; (ii) intensity score based on three levels—1 is low, 2 is intermediate and 3 is strong (see Figure 1); and (iii) Allred score is the sum of the proportion of positive tumor cells and the intensity of immunostaining in those cells, giving a final score of 0 (negative) or between 2 and 8. On the other hand, F-score and subjective measures were used to assess the response of this work in the classification and quantification process. The ICC was used to estimate inter-rater reliability on quantitative data because it is highly flexible [35]. The ICC inter-rater agreement measures were interpreted following guidelines by Terry [36]: (i) Less than 0.50, poor; (ii) between 0.50 and 0.75, moderate; (iii) between 0.75 and 0.90, good; and (iv) between 0.90 and 1.00, excellent.

### 2.1. Evaluation of Block-Based *Wnt-1* Segmentation

**Figure 2:**
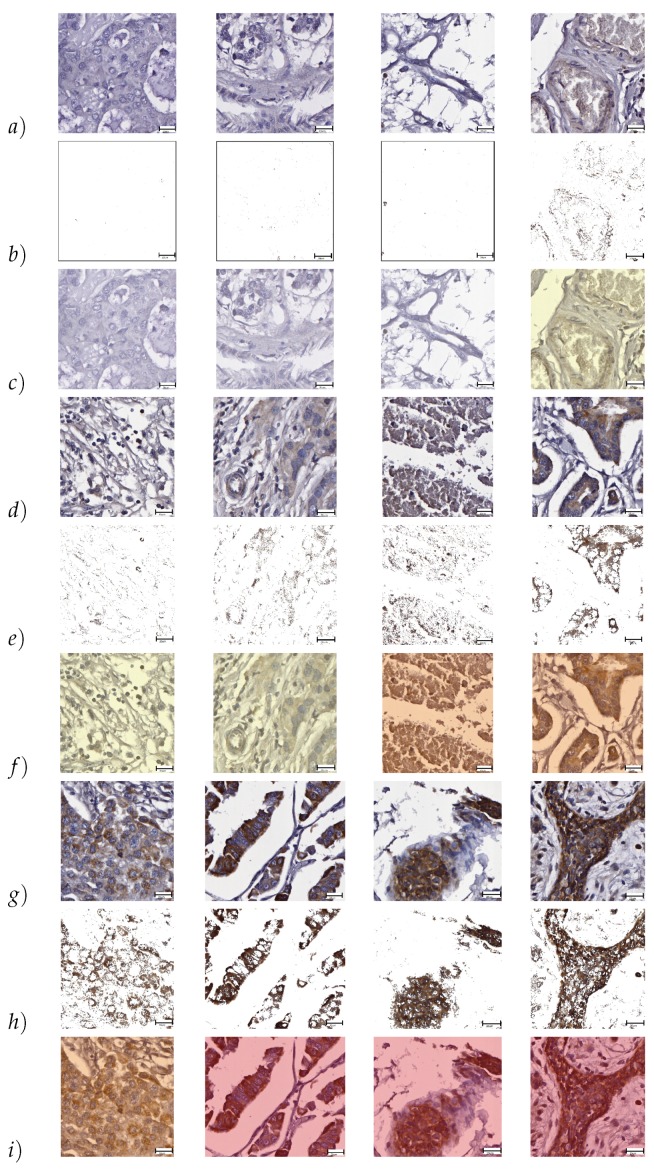
Selected *Wnt-1* segmentation and classification results: (**a**,**d**,**g**) original image blocks are presented; (**b**,**e**,**h**) automatic segmentation using color convolution; and (**c**,**f**,**i**) automatic Allred score classification, scale bar = 20 μm.

A selected set of image blocks of different TMA tissue core images, *Wnt-1* segmentation results and *Wnt-1* classification according to Allred score is shown in Figure 2. Classification results are represented using watermark with distinctive colors as follows: (i) white represents [0–1] Allred score; (ii) yellow represents [2–3] Allred score; (iii) orange represents [4–6] Allred score; and (iv) red represents [7–8] Allred score.

**Figure 3:**
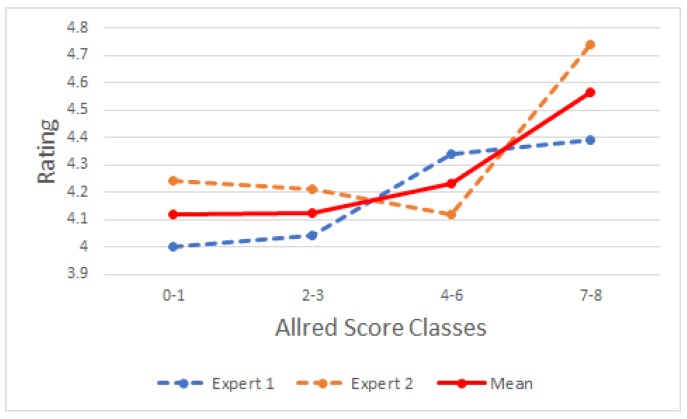
Results obtained from *Wnt-1* positive segmentation. The y-axis is not displayed from the origin to improve visualization.

Results of the automated *Wnt-1* segmentation were evaluated by two experts and using a scale from 1 to 5 to represent poor, average, good, very good, and excellent. The *Wnt-1* TMA staining was further reviewed by two pathologists (I.Z. and M.V.), whose analysis was compared to the final automated staining data. Figure 2 shows that *Wnt-1* staining is absent from cell nuclei, consistent with *Wnt-1* being a cytoplasmic biomarker. Figure 3 contains a graphical representation of the mean of the evaluations of the ability of the approach to recognize *Wnt-1* positive cells in the set of test block images.

The ability of the proposed approach to recognize *Wnt-1* was given an average score of 4.26 by the experts. The highest average was obtained for the [7–8] class and the lowest average was obtained for the [0–1] class. This lower score is due to the potential confusion between *Wnt-1* and artifactual staining and/or debris and signal in the stroma that had not been removed. An advantage of the approach used is the segmentation of small *Wnt-1* positive areas—sometimes imperceptible to the eye—and the possibility to evaluate the complete sample with pixel precision.

### 2.2. Evaluation of Block-Based *Wnt-1* Classification

**Figure 4:**
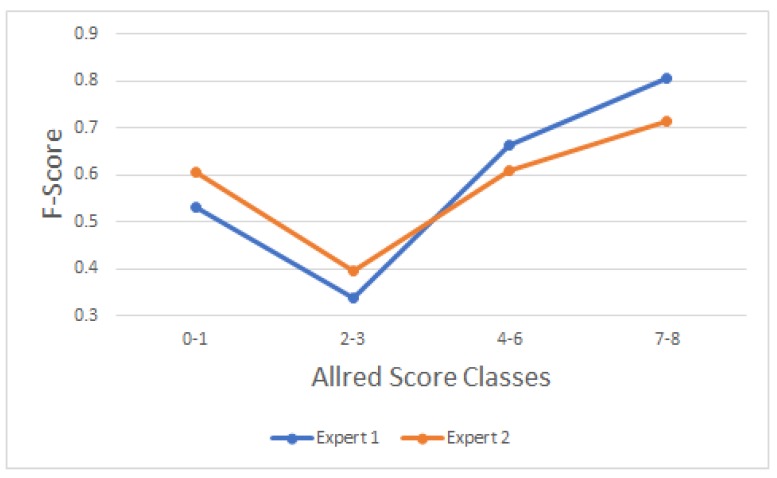
F-Score obtained with *Wnt-1* automatic classification using Expert-1 and Expert-2’s Allred score. [0–1] represents no affect, [2–3] represents small, [4–6] represents moderate, and [7–8] represents good. The y-axis is not displayed from the origin to improve visualization.

The classification results are shown in Figure 2. The confusion matrices [37] of automatic *Wnt-1* classification, with each expert’s evaluation, for four classes using Allred classification—[0–1], [2–3], [4–6] and [7–8]—are presented in Table 1 and Table 2, respectively. Additionally, a graphical representation of F-score measures is illustrated in Figure 4. The results obtained were calculated using the set of block test images. The results obtained yield between 0.338 and 0.804 F-score using our approach. The highest F-score was obtained for the [7–8] class in both cases. The lowest F-scores were achieved for the [2–3] class in both cases, owing to its high similarity with the [4–6] class, taking into account the presence of non-tumor cells, apoptotic nuclei and debris. ICCs of 0.634, 0.791, 0.551 and 0.630 were obtained for the classes [0–1], [2–3], [4–6] and [7–8], respectively.

Analysis of the confusion matrices shows that high risk classes were correctly classified, while low risk classes presented some confusion with adjacent classes. This is due to the similarity between adjacent classes. Nevertheless, the results illustrate the extent to which the block-based automatic classification matches manual scoring.

### 2.3. Evaluation of *Wnt-1* Classification in a TMA Tissue Core Image

**Figure 5:**
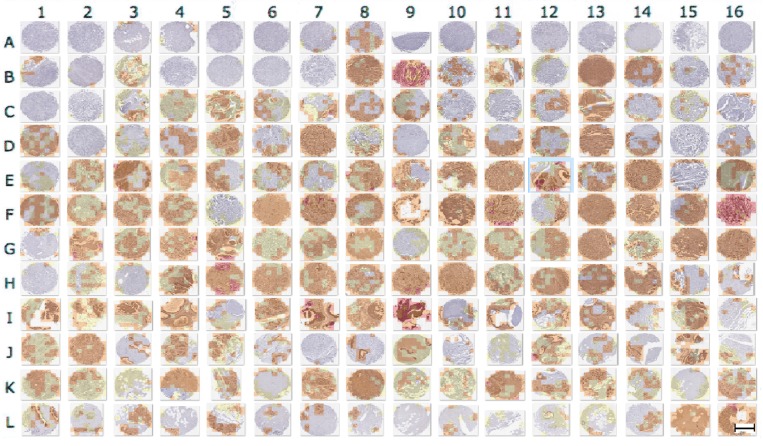
Results obtained for *Wnt-1* positive classification in complete TMA tissue core images, scale bar = 20 μm.

Complete TMA results were obtained considering the TMA information, the result obtained (see Figure 5) and expert evaluation. We calculated the average ICC obtained as 0.752, showing a moderate degree of agreement between the evaluators and the automated classification.

## 3. Discussion

Color deconvolution is a robust and flexible method to identify and separate the DAB signal of the stain used. Color deconvolution and its variants have been used successfully in different histological and histopathological applications [38], showing advantages in determining staining densities, ratios and even for recognizing different structures.

**Figure 6:**
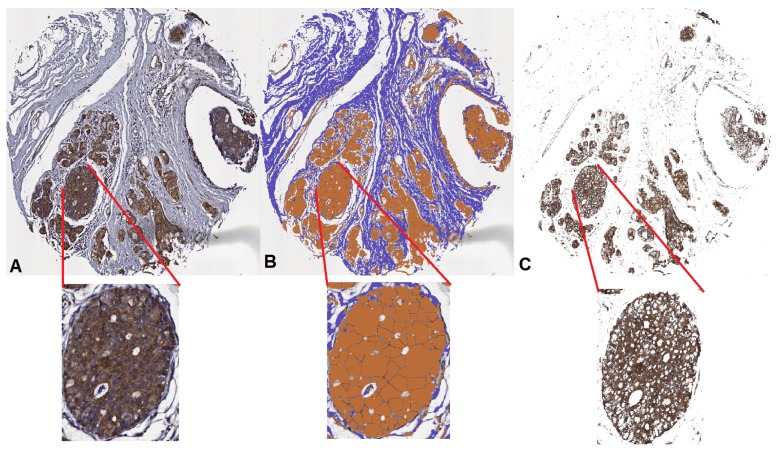
Comparison between immunoRatio and NUBIA*Wnt-1*1Ratio: (**A**) original TMA tissue core image; (**B**) ImmunoRatio results; and (**C**) NUBIA*Wnt-1*1Ratio results.

The results obtained cannot be compared directly to other approaches in the literature because, although there are other solutions for automated biomarker identification, these use proprietary software [21] or are based on nuclear biomarkers [18,20]. Nonetheless, AQUA uses immunofluorescence intensity data to measure expression and, in some cases, is supported by additional information such as sub-cellular localization. AQUA has been used to measure several markers, including EGFR, ER, mTOR and PTEN. We compared the algorithm proposed in [18], called ImmunoRatio, with our method. Figure 6 illustrates the contrast among methods and shows that ImmunoRatio: (i) includes cell nuclei in biomarker identification; and (ii) separates particles trying to simulate cell nuclei in DAB areas.

Our proposed method is based on dividing the image into image blocks, using color deconvolution and a K-means algorithm. Experimental evaluation has shown that our approach identified and quantified *Wnt-1* levels in a similar way as an approach that would be used in the clinic.

## 4. Materials and Method

### 4.1. Problem Statement

**Figure 7:**
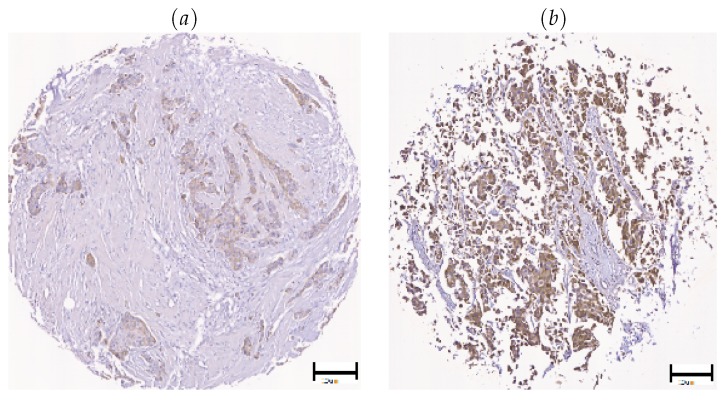
Illustration of stained cases of *Wnt-1*: (**a**) heterogeneously; and (**b**) homogeneously, scale bar = 20 μm.

**Figure 8:**
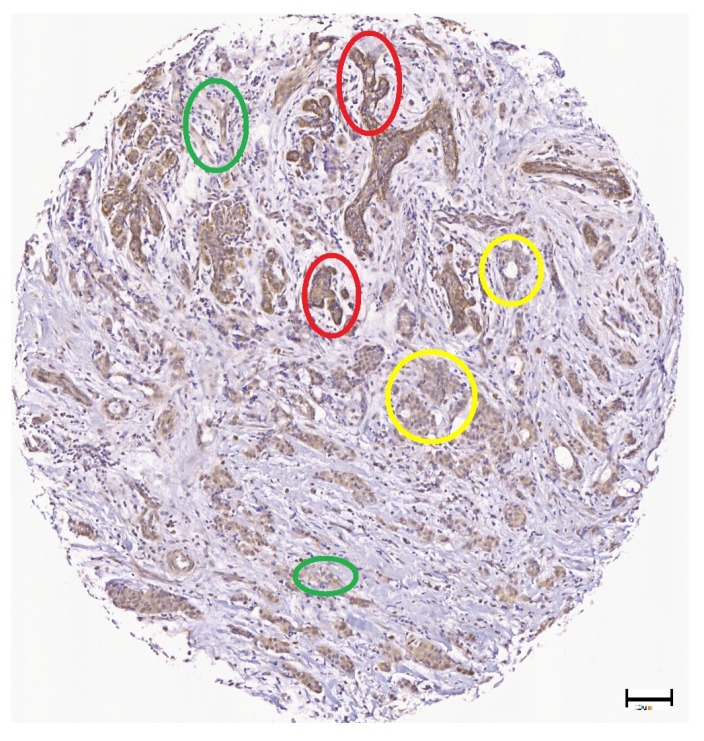
Illustration of *Wnt-1* intensity level variation. The red circles correspond to high intensity level examples, the yellow circles correspond to medium level examples and the green circles correspond to low intensity level examples, scale bar = 20 μm.

The level of expression of *Wnt-1* may be a biomarker for some breast cancers. However, *Wnt-1* expression may vary in the same sample, according to the selected region of interest in the image (see Figure 7) or the level of *Wnt-1* expression in the patient sample (see Figure 8). Color is the most important feature to analyze IHC in histopathology images [39]. One of the most relevant problems is that environmental factors and image acquisition devices can affect image quality and automated results. We used a TMA that was captured using an Aperio Digital Pathology Slide Scanner to achieve high quality images and reduce thermal drift and colour balance. There are also other types of variation in sectioning and staining processes and our approach increases robustness using color deconvolution and a clustering algorithm. Clinical researchers often use the Allred score to score samples manually. Allred score considers two aspects: (i) the proportion of cells that are positive over the evaluated area; and (ii) the intensity level of the positive staining. Pathologists normally examine larger areas or multiples sections to confirm their observations. The details of the complete process are presented in the following section.

**Figure 9:**
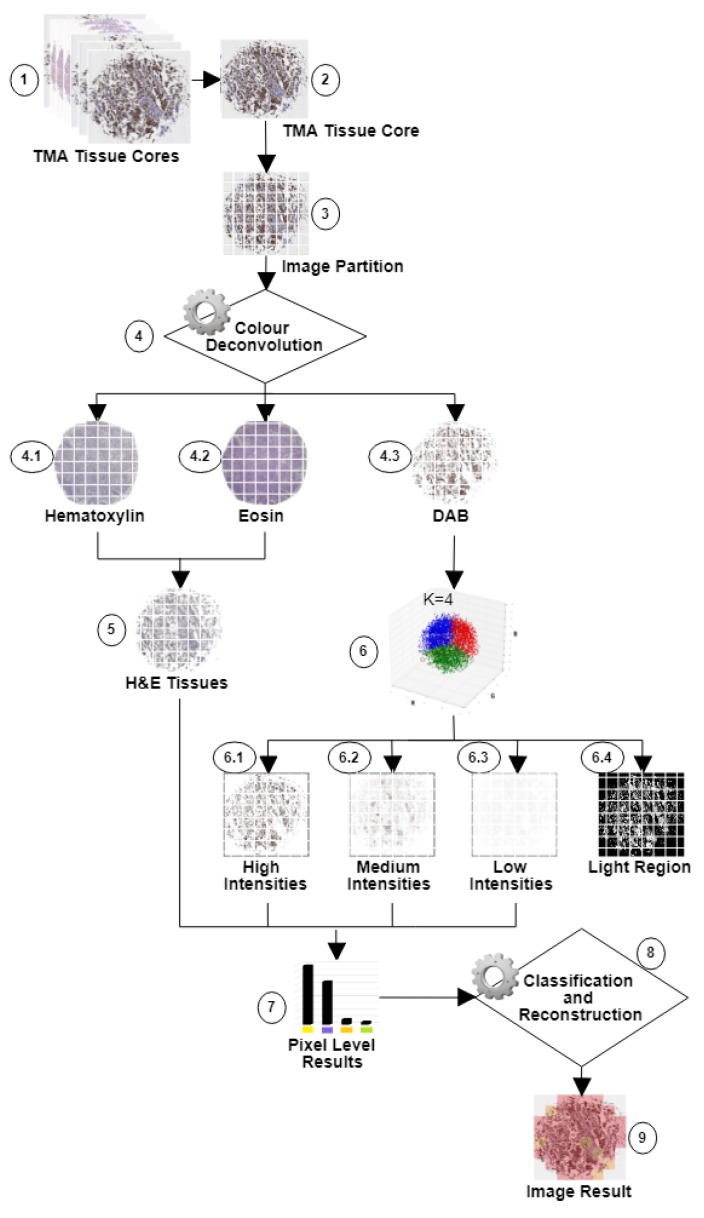
Proposed approach for automated segmentation and quantification of *Wnt-1*: (1) TMA tissue core images with DAB (brown), hematoxylin (blue) and eosin (magenta). (2) TMA tissue core image. (3) 64 image blocks. (4) Color Deconvolution process. Color Deconvolution results: (4.1) hematoxylin, (4.2) eosin and (4.3) DAB of the original image. (5) Tissues without DAB. (6) Clustering process with K-means algorithm using initial labels k=4. (6.1) *Wnt-1* high intensities. (6.2) *Wnt-1* medium intensities. (6.3) *Wnt-1* low intensities. (6.4) Light regions. (7) Quantification results at pixel level. (8) Classification and reconstruction using Allred score. (9) Image result. In the resulting images, the black or color regions correspond to segmented areas.

Using *Wnt-1* as an example, we propose a method for in silico classification and quantification of a cytoplasmic biomarker in breast cancer. A brief summary of the proposed process is given below and a detailed explanation is presented in the following subsections. Our method is composed of four steps: (i) a TMA tissue core image is divided into blocks; (ii) DAB areas are identified for each block using color deconvolution; (iii) DAB areas are classified using K-means algorithm by blocks; and (iv) each core image is classified using block-based classification. Figure 9 shows a general outline of our approach. (1) A set of TMA tissue core images are obtained from one or many TMAs. (2) Each TMA tissue core image is processed. (3) Blocks of m×n pixels are obtained from the input image. In this study, 64 blocks by image were obtained. (4) After evaluating different methods to identify DAB, color deconvolution is used to separate: (4.1) Hematoxylin; (4.2) Eosin; and (4.3) DAB. (5) Pixels that represent tissues are identified using (4.1) and (4.2). A pixel position is represented by a three-element feature vector Red, Gree and Blue (RGB) representing the amount of each colour in that position contains. (6) The input for the K-means algorithm is composed by the set of RGB vectors for the DAB image and the *K* parameter, representing the number of clusters to obtain, which has been set to four groups: (6.1) high *Wnt-1* positive intensity levels; (6.2) medium *Wnt-1* positive intensity levels; (6.3) low *Wnt-1* positive intensity levels; and (6.4) light regions (background and spaces between tissues). The black regions correspond to segmented areas. (7) Tissues and DAB proportion measures are calculated. (8) Blocks are classified by Allred score and then segmentation of the entire image is formed by stitching classified blocks. (9) A classified TMA tissue core image is obtained. The details of the complete process are presented in this section.

### 4.2. Experimental Setup

Tissue specimen sample cores in the breast TMA were immunostained for the *Wnt-1* protein. The TMA was composed of 192 tissue cores, we used one image per tissue core, and 12,288 image blocks were used for validation (70%) and testing (30%). The images were acquired with variable pixels of resolution according to each tissue core, between 3968×4970 and 5400×5500, and the images were stored in JPG format. To avoid introducing additional margins of error, the images were not modified further. Block sizes varied according to the pixel resolution of each TMA core image, minimizing differences between blocks. The datasets belonging to image blocks obtained from different samples and patients were acquired using a 20× objective. Two scientists familiar with immunohistochemical analysis of TMAs reviewed the TMA tissue core images blindly and graded the cytoplasmic staining for *Wnt-1* intensity and percentage of positive cells, according to the Allred scoring method. An Aperio Digital Pathology Slide Scanner with eyepieces with a magnification factor of 10× and a field of view of 20 obtaining 200 end magnification for a 20× objective was used. We have made the datasets publicly available at: https://vicomtech.box.com/v/Wnt1Dataset. Algorithms were implemented in Python, using the OpenCV library for computer vision [40], on a computer with 4 cores, 8 GB memory and a NVIDIA Titan X Pascal GPU.

### 4.3. Partitioning TMA Tissue Cores into Blocks

Our approach is to identify *Wnt-1*-positive areas using a block-based strategy. A block is the analysis unit to identify, classify and quantify *Wnt-1*-positive areas in an image. A block is a fixed non-overlapping m×n partition of a TMA tissue core image. The block size depends on the original image size; 64 blocks are obtained per image. The number of blocks was decided heuristically taking into account that, if the block size is too small or too large, high variations may hinder its analysis.

### 4.4. Block-Based *Wnt-1* Segmentation

**Figure 10:**
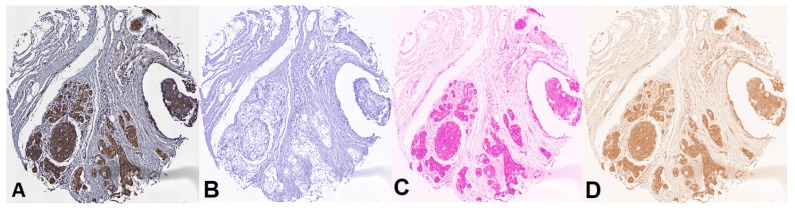
Colour deconvolution in histopathological images: (**A**) original TMA tissue core image; and (**B**–**D**) color deconvolution results separating the contribution of hematoxylin (**B**), eosin (**C**) and DAB (**D**) to the original image. Magnification 20×.

Color information is a discriminant feature for IHC staining analysis. We use the color deconvolution strategy proposed in [41]. This method is based on orthonormal transformation of the original RGB image of samples stained with H&E and DAB at different staining levels. This method is composed of two steps: (i) color representation; and (ii) color deconvolution. The method proposed in [41] provides a robust and flexible method for objective IHC analysis of samples; it provides the possibility to determine staining densities even in areas where multiple stains are co-localized, making it possible not only to determine surface area and overall absorption in areas with a specific colour, but also to determine densities and ratios of densities of stains in each area (see Figure 10).

### 4.5. Block-Based *Wnt-1* Classification

In this proposal, we classify blocks into four classes at pixel level—high intensity, medium intensity, low intensity and light regions—using the K-means algorithm with k=4. The K-mean’s inputs are the initial centers—during the first attempt, we used the user-supplied labels instead of computing them from the initial centers—and the RGB values. Initial centers were established heuristically taking into account the expert’s evaluation for intensity level of *Wnt-1* positive.

A laboratory protocol and an image capture protocol were defined to have an image dataset with similar characteristics and to reduce errors in the automatic evaluation. However, thermal drift and color balance affect the analysis introducing small variations in the intensities of the colors and lighting and other variations may be introduced during the tissue staining process. This situation implies that the proposed method has to be robust to small variations in color balance and thermal drift that may occur. Invariance is granted by the K-means algorithm and the protocols.

Let $I:I\times I\to R^{3}$ be a block of size m×n pixels in RGB color space; $H_{k}\left(t\right)$ is a cluster represented by a set of vectors in $R^{3}$ in the *t*th iteration; and $C_{k}\left(t\right)\in R^{3}$ be a centroid *K* of the cluster $H_{k}\left(t\right)$. We use RGB values since they contain relevant information about IHC and H&E. The initial parameters of the K-means algorithm are set: t=0, $C_{1}\left(0\right)=\left\{64,32,21\right\}$, $C_{2}\left(0\right)=\left\{105,51,27\right\}$, $C_{3}\left(0\right)=\left\{124,87,45\right\}$, $C_{4}\left(0\right)=\left\{255,255,255\right\}$. We write K-means in two steps: (1) assignment step where each pixel $I_{ij}$ is assigned to the cluster $H_{k}$ which centroid $C_{k}$ is the closest in the Euclidean way:

(1) $$I_{ij}\in H_{k}\left(t\right)\;\mathrm{if}\;k=\underset{k\in \{1,2,3,4\}}{arg\;min}\left|\right|I_{ij}-C_{k}\left(t\right)\left|\right|;$$

and (2) an update step where each centroid $C_{k}$ is updated based on the observations that belong to its cluster $H_{k}$:

(2) $$C_{k}\left(t+1\right)=\frac{1}{|H_{k}\left(t\right)|}\sum_{i=1}^{M}\sum_{j=1}^{N}a_{ij}.I_{ij},$$

where $a_{ij}$ is $I_{ij}$ if $I_{ij}\in H_{k}\left(t\right)$ and 255 in other case. These two steps are carried out iteratively until convergence. Let $O=\{O_{1},O_{2},O_{3},O_{4}\}$ be a image in RGB such that:

(3) $${\left(O_{k}\right)}_{ij}=\left\{\begin{array}{cc} I_{ij} & \;I_{ij}\in H_{k}\left(t\right)\\ \left\{255,255,255\right\} & \;\mathrm{else}, \end{array}\right.$$

where a value of *k* represents a different class in *I*, such that k=1 corresponds to high DAB intensities pixel positions (associated with cytoplasm), k=2 corresponds to medium DAB intensities, k=3 corresponds to low DAB intensities, and k=4 corresponds to light regions. Thus, we obtain DAB levels in an image block with the RGB values of *I*.

### 4.6. *Wnt-1* Classification in a TMA Tissue Core Image

*Wnt-1* classification in a TMA tissue core image using the block-based *Wnt-1* classification (see Figure 9, Steps (8) and (9)) is defined as:

Let $I:I\times I\to R^{3}$ be a TMA tissue core image in RGB colour space; $I_{s}=\left\{I_{0},I_{1},...,I_{K}\right\}$ be a TMA or a set of TMAs; *B* be a matrix of blocks in which each $B_{ij}$ represents the *j*th block of the image *i*; $M(B_{ij})$ be the block-based *Wnt-1* classification method; $Wc_{I}$ be a m×m matrix of labels where m=8. Then, *Wnt-1* classification of a TMA tissue core image using block-based recognition is:

(4) $$Wc_{I}=\left(\begin{array}{cccc} M(B_{11}) & M(B_{12}) & \cdots & M(B_{1m})\\ M(B_{21}) & M(B_{22}) & \cdots & M(B_{2m})\\ \vdots & \vdots & \cdots & \vdots \\ M(B_{m1}) & M(B_{m2}) & \cdots & M(B_{mm}) \end{array}\right).$$

## 5. Conclusions

In this paper, we present an in silico approach that allows the classification and quantification of a cytoplasmic protein in breast cancer histopathological images with an average F-score and accuracy greater than 0.58% and 97% according to the class of risk to identified, being more precise for the high risk classes ([7–8] Allred Score).

High variability between expert’s evaluations are due to the subjective criteria used—proportion and intensity. The misclassified classes resulted from additional features, including the proportion of tumor cell cells and signal from stromal cells and apoptotic cells. However, average ICC was improved with the proposed approach.

Using the recognized *Wnt-1* positivity from a block of size m×n, we were able to classify it according to Allred score. Taking into account these results, it is possible to classify TMA tissue core images by extracting the appropriate segmentation with the selection of the proper classifier. Color deconvolution is a robust and flexible method that determines density and ratios of densities of stains in each area. In addition, the proposed in silico approach is faster than the traditional manual approach.

Using markers such as *Wnt-1* may in future identify breast cancer patients with a high risk of tumor recurrence and/or progression to metastasis, who may then benefit from further intensive therapy after a surgery [6].

We have created and made publicly available a dataset consisting of 12,288 image blocks—192 TMA tissue cores images—that can be used to validate the results obtained in our work or to improve upon the proposed method.

In the future, we will extend this proposal through the following five lines of investigation: (i) develop an approach that excludes stromal cells and return a classification by tissue core; (ii) integrate our approach with other cytoplasmic and nuclear biomarkers (e.g., *Ki-67*, *ER*, *PR*, and *Sox2*); (iii) evaluate ROIs with different shapes; (iv) explore new classification techniques, such as deep learning algorithms; and (v) compare our proposal with other approaches in the literature.

## Figures and Tables

**Table 1:** Confusion Matrix of Automatic Classification Based on Allred Score with Expert-1. The best F-score value is presented in bold.

	[0–1]	[2–3]	[4–6]	[7–8]	F-Score
[0–1]	38	5	0	0	0.531
[2–3]	62	34	5	0	0.338
[4–6]	0	54	88	23	0.664
[7–8]	0	7	7	76	**0.804**

**Table 2:** Confusion Matrix of Automatic Classification Based on Allred Score with Expert-2. The best F-score value is presented in bold.

	[0–1]	[2–3]	[4–6]	[7–8]	F-Score
[0–1]	47	7	1	0	0.606
[2–3]	47	37	3	0	0.396
[4–6]	6	53	86	37	0.610
[7–8]	0	3	10	63	**0.716**

## Abbreviations

The following abbreviations are used in this manuscript:

DAB	Diaminobenzidine
H&E	Hematoxylin and Eosin
TMA	Tissue Microarray
ICC	Intraclass Correlation
IHC	Immunohistochemistry
MMTV	Mouse Mammary Tumour Virus
CSC	Cancer Stem Cell
ER	Estrogen Receptor
ER+	Estrogen Receptor-positive
PR	Progesterone Receptor
DIA	Automated Digital Analysis
AQUA	Automated Quantitative Analysis
RGB	Red, Green and Blue
